# Fitting hidden Markov models of protein domains to a target species: application to *Plasmodium falciparum*

**DOI:** 10.1186/1471-2105-13-67

**Published:** 2012-05-01

**Authors:** Nicolas Terrapon, Olivier Gascuel, Éric Maréchal, Laurent Bréhélin

**Affiliations:** 1 Méthodes et Algorithmes pour la Bioinformatique, LIRMM, Univ. Montpellier 2, France; 2 Institute für Evolution and Biodiverstät – Westfälische Wilhelms-Universität, Germany; 3 CEA Grenoble iRTSV/LPCV,17 rue des Martyrs, France

## Abstract

**Background:**

Hidden Markov Models (HMMs) are a powerful tool for protein domain identification. The Pfam database notably provides a large collection of HMMs which are widely used for the annotation of proteins in new sequenced organisms. In Pfam, each domain family is represented by a curated multiple sequence alignment from which a profile HMM is built. In spite of their high specificity, HMMs may lack sensitivity when searching for domains in divergent organisms. This is particularly the case for species with a biased amino-acid composition, such as *P. falciparum*, the main causal agent of human malaria. In this context, fitting HMMs to the specificities of the target proteome can help identify additional domains.

**Results:**

Using *P. falciparum* as an example, we compare approaches that have been proposed for this problem, and present two alternative methods. Because previous attempts strongly rely on known domain occurrences in the target species or its close relatives, they mainly improve the detection of domains which belong to already identified families. Our methods learn global correction rules that adjust amino-acid distributions associated with the match states of HMMs. These rules are applied to all match states of the whole HMM library, thus enabling the detection of domains from previously absent families. Additionally, we propose a procedure to estimate the proportion of false positives among the newly discovered domains. Starting with the Pfam standard library, we build several new libraries with the different HMM-fitting approaches. These libraries are first used to detect new domain occurrences with low E-values. Second, by applying the Co-Occurrence Domain Discovery (CODD) procedure we have recently proposed, the libraries are further used to identify likely occurrences among potential domains with higher E-values.

**Conclusion:**

We show that the new approaches allow identification of several domain families previously absent in the *P. falciparum* proteome and the Apicomplexa phylum, and identify many domains that are not detected by previous approaches. In terms of the number of new discovered domains, the new approaches outperform the previous ones when no close species are available or when they are used to identify likely occurrences among potential domains with high E-values. All predictions on *P. falciparum* have been integrated into a dedicated website which pools all known/new annotations of protein domains and functions for this organism. A software implementing the two proposed approaches is available at the same address: http://www.lirmm.fr/∼terrapon/HMMﬁt/

## Background

Among the annotations that can be attached to a protein, domains occupy a key position. Protein domains are sequential and structural motifs that are found independently in different proteins, in different combinations. As such, domains seem to be functional subunits of proteins above the raw amino acid sequence level [1]. Several approaches have been developed to define and identify protein domains. Some are based on a structural classification scheme [2], while others are inferred by clustering conserved sub-sequences [3,4]. One of the most widely used domain schemata is the Pfam database [4]. In Pfam, each domain family is defined using a set of distinct representative protein sequences which are manually selected and aligned, and used to learn a Hidden Markov Model (HMM) [5] of the domain. HMMs are probabilistic models which use *match* states to model the conserved positions of the multiple sequence alignment, and handle the gaps with specific (*insert* and *delete*) states.

The Pfam database (version 23.0) offers a collection of 10 340 HMMs/domains, which cover over 73% of all proteins in the Uniprot database [6]. The InterPro consortium [3] has functionally annotated a subset of Pfam HMMs using the Gene Ontology (GO) [7]. According to the InterPro annotation policy, a domain is annotated with a given GO term if all proteins where this domain is known also share this GO term. This stringent rule allows, when a new domain is detected in a protein, to transfer its annotations to this protein. Enhancing domain detection is thus a fundamental step for improving structural and functional annotations of proteins.

When analyzing a new protein sequence, each Pfam HMM is used to compute a score that measures the similarity between the sequence and the domain. If the score is above a given threshold provided by Pfam (score thresholds differ depending on the HMMs), then the presence of the domain is asserted in the protein. This threshold is referred to as the *gathering threshold* and is manually curated to ensure few false positives among detected domains [4] However, when applied to highly divergent proteins, this strategy may miss numerous domains. This is the case with *Plasmodium falciparum*, the main causal agent of human malaria, which kills nearly 800 000 people each year among the 106 malaria-endemic countries [8]. No Pfam domains are detected in nearly 50% of *P. falciparum* proteins, while many domain types seem to be missing from its repertory. Although this situation may be explained by the existence of genes that are unique to this organism, it is further exacerbated by the high evolutionary distance between *P. falciparum* and the classical model organisms that were used to build the HMMs. Accurately estimating the number of Pfam domains that remain to be discovered in *P. falciparum* is challenging. In classical model Eukaryotes, the number of Pfam occurrences per proteins is above 0.8 (for example the coverage of *S. cerevisiae* and *C. elegans* is 0.9 and 0.86, respectively). Assuming a coverage of 0.8, a total ∼4500 Pfam occurrences should be present in the proteome of *P. falciparum*. Subtracting the number of currently annotated domains from the expected 4500 would suggest that around 1 000 domains are yet to be detected. These “missing” occurrences might be explained by the highly atypical genome of *P. falciparum*, which is composed of above 80% A+T, and involves long low-complexity insertions of unknown function believed to form non-globular domains [9]. This strongly biases the amino-acid composition of *P. falciparum* proteins, in which six amino acids account for more than 50% of the protein composition [10]. In this context, fitting the HMM library to the specificities of the target proteome may help identify additional domains not detected by the standard library.

To the best of our knowledge, two studies address this problem. First, an *a posteriori* correction of domain scores has been introduced by Coin *et al.*[11]. This correction takes the prior probability of each domain family in the target species into account. Prior probabilities are estimated using asserted domain occurrences in the closest relatives of the species. A second approach is to build taxon-specific models by integrating known domain occurrences from the nearest species into the multiple sequence alignment. For example, this method has been successfully applied to fungi by Alam *et al.*[12] thanks to the availability of 30 fungal genomes. However, both approaches have an obvious drawback: they can only discover new occurrences of domain families already asserted in the target or its closest relatives.

Here, we propose two new approaches to circumvent this limitation by correcting the entire HMM library. The principle of these approaches is to learn overall correction rules which are applied to the emission probabilities of the match states of all HMMs. In the first approach, an amino-acid substitution matrix dedicated to the target organism is estimated and applied to the emission probabilities of the match states to mimic the evolution toward the amino-acid composition of the target species. Our second approach involves partitioning all match states of the Pfam library in clusters with similar amino-acid emission probabilities, and to use the known domain occurrences in the target species to learn specific correction rules for each class of match state.

Once a new HMM library has been built, it is used to detect new domain occurrences with low E-values. As explained above, the original Pfam library provides, with each HMM, a manually curated threshold which ensures very low false positive rates among the detected domains. However, after HMM correction, these thresholds can no longer be safely used. We propose a simple approach to estimate the False Discovery Rate (FDR) of the newly discovered domains of each corrected library. This procedure enables us to compare the results achieved by each correction method at equivalent FDR.

In the following, we first review the previously described approaches to fit an HMM library to a target species. We describe our own approaches, and present the statistical procedure for FDR estimation. The four correction methods are used to detect new domain occurrences in the *P. falciparum* genome. In these experiments, we distinguish two cases depending on whether genomes close to the target organism are available or not. Finally, we use the corrected libraries to find additional domains with the Co-Occurrence Domain Discovery (CODD) procedure we have recently proposed [13]. This procedure identifies divergent domain occurrences on the basis of co-occurrence properties, and uses its own procedure to estimate FDRs associated with the results. All predictions achieved with the corrected libraries have been integrated into a dedicated website and can be browsed at http://www.lirmm.fr/∼terrapon/HMMﬁt/. A program implementing the two proposed approaches is available at the same address.

## Method

In the following, the target species is denoted as *s*. The *known domain occurrences* of a species are all domain occurrences identified with the original Pfam library using the recommended thresholds.

### Previous approaches

To the best of our knowledge, two approaches, summarized below, have been proposed to fit an HMM library to a target species.

#### Score adjustment

Coin *et al.*[11] proposed an *a posteriori* correction of the score function of HMMs. In this solution, the correction does not involve HMMs but rather the function used to score domain occurrences. This is done by incorporating information about the specific domain-frequency of the target organism. The adjusted domain scores (log odds ratio) are obtained by adding the term *log*(*P*(*d*|*s*)/*P*(*d*)) to the original score function, where *P*(*d*) denotes the prior probability of domain *d* (*i.e.* its probability in Uniprot), and *P*(*d*|*s*) denotes the corresponding probability in the target organism. This correction increases the score of domains that are more likely in the species, and decreases the score of other domains. *P*(*d*|*s*) is estimated from a weighted average of the frequency of *d* in the already known domains of *s* and in that of the other sequenced species of its genus, class, phylum and kingdom. This is done according to the recursive formula

(1) $$P(d|S_{i})=0\cdot 5\cdot \frac{n(d,S_{i})}{n_{\mathrm{tot}}(S_{i})}+0\cdot 5\cdot P(d|S_{i+1}),\text{for}i=m-1\text{to}0,$$

where *S*_0_ is the target organism *sS*_*i*_ is the *i*th parent taxon, *S*_*m*_ is the kingdom, *n*(*dS*_*i*_) is the number of domains *d* in taxon *S*_*i*_, and *n*_*tot*_(*S*_*i*_) is the total number of domains in *S*_*i*_. For *i*=*m* we use $P(d|S_{m})=\frac{n(d,S_{m})}{n_{\mathrm{tot}}(S_{m})}$.

#### Enriched alignments

A simple approach to fit an HMM to a target species is to enrich the alignment used to learn the HMM with known domain occurrences in the species or its close relatives (see for example Alam *et al.*[12]). Given an HMM library and a set of protein sequences from the closest species of the target organism, the procedure involves: 

1. identifying all domain occurrences in the selected species using the original library with recommended thresholds,

2. building new multiple sequence alignments by integrating the identified domains into the original alignments using the Viterbi algorithm [5],

3. building new HMMs from new alignments using HMMER software [14].

### Two new approaches

Both previous approaches have the same drawback: only domain families already known in the target or its close relatives may benefit from detection improvement. We propose to circumvent this limitation by learning global correction rules that are applied to match states of all HMMs, in order to build a new and complete adapted library. We focus on match states because they model the conserved positions of the domains and thus bring most of the information contained in the alignments. The challenge is to derive rules that simulate the drift that separates the target organism sequences from the sequences used to train the HMM, while preserving as much position-specific information of the HMMs as possible. We propose two approaches for this, that are described below. In the following, *X* denotes the set of all match states of all HMMs. Each state *x*∈*X* is described by a vector (*x*_*i*_)_*i*∈[1*..*20]_where *x*_*i*_ is the probability to emit the amino acid *i* in state *x*.

#### Substitution matrix

Substitution matrices (*e.g.* JTT [15], WAG [16]) are essential for computing substitution probabilities along branches in phylogenies, and thus for computing the likelihood of the data [17]. We distinguish the substitution rate matrix containing the instantaneous rates of change from any amino acid to another, and the substitution probability matrix which contains the probabilities of change from one amino acid to another when elapsed time is *t*. These matrices are associated with a stationary distribution of amino acids; when *t* is large enough, the probability of any amino acid tends towards its stationary probability, regardless of the initial probability distribution. Here, we use this property to move the amino-acid distribution of match states toward the distribution of the target species, thus simulating the evolution of conserved positions under the constraints characteristic of this species.

Let *Q*=(*q*_*ij*_) be the rate matrix and *P*(*t*)=(*p*_*ij*_(*t*)) be the probability matrix associated with *t*, where *i* and *j* denote two amino acids and *ij* the change from *i* to *j*. We use a general time reversible (GTR) model, and thus *Q* is composed of two parts: the stationary amino-acid distribution *π*=(*Π*_*i*_) and the symmetric exchangeability matrix *R*=(*r*_*ij*_), using equality: *q*_*ij*_=*r*_*ij*_*Π*_*j*_ (*i*≠*j*). The diagonal elements are such that the row sums are all zero. *Q* is normalized, that is $-\underset{i}{\sum }\Pi_{i}q_{\mathrm{ii}}=1$, so that an evolutionary time *t* of 1 corresponds to an expected number of substitutions per site equal to 1. Here we use the *R* matrix of the LG model [18], which refines previous general purpose models such as JTT or WAG, and a *π*distribution computed from *P. falciparum* proteins (see below for details). *P*(*t*) is obtained by matrix exponentiation: *P*(*t*)=^*eQt*^.

The new HMM library (for given *t*) is built by applying *P*(*t*) to the probability distributions of all match states (of all Pfam HMMs) using equation: *x*∗=*x*·*P*(*t*), where *x* is the original distribution vector of the match state, *x*∗ the new one, and both are row vectors. The evolutionary time *t* has to be carefully chosen: if too large, then all *x*∗ distributions tend toward *π* and are uninformative; if too small, then *x*∗∼ *x*and the correction has no effect. Several values ranging from 0.01 to 0.5 were tested in our experiments (see below).

#### Match-state clustering

**Figure 1:**
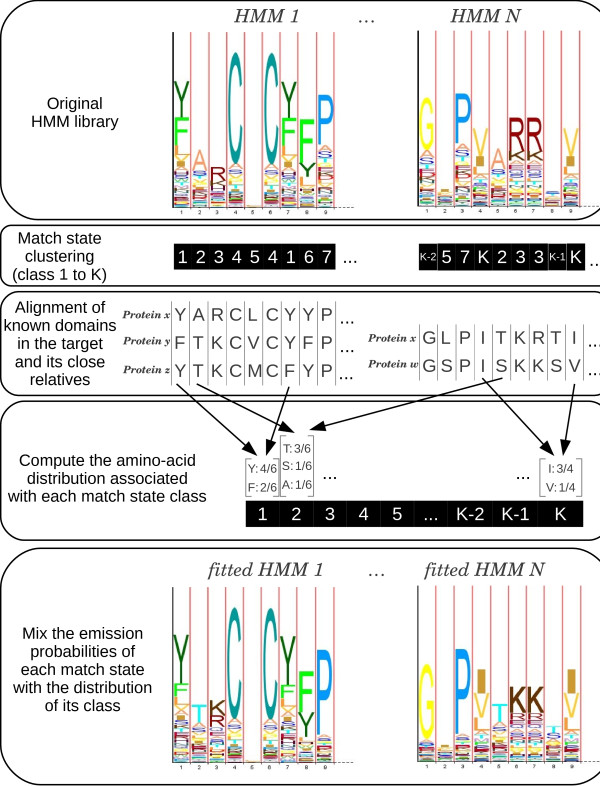
**The match-state clustering approach.** We start with a library of *N* HMMs. First, the match states are clustered into *K* classes according to their amino-acid distribution. Next, the known domain occurrences of the target species and its close relatives are aligned to the states of the corresponding HMMs. These alignments are used to compute an amino-acid distribution for each state class. For example, if we suppose that class 2 only involves the second state of HMM 1 and the 5th state of HMM *N*, the distribution estimated from the represented alignments is 3/6 for T, 1/6 for S, 1/6 for A, and 0 for all other amino acids. Finally, the new HMM library is built by mixing the original distribution of each match state with the estimated distribution of its class.

In profile HMMs, match states model the amino-acid distribution of conserved positions. This distribution reflects the physical and chemical constraints associated with the position. Thus, positions with similar distribution are likely to undergo similar constraints. The principle of the second approach is to cluster the match states of HMMs according to their amino-acid distribution, and then to learn correction rules for each of the cluster of match state (see Figure 1). First, the *K*-means algorithm [19] is applied on the set *X*. This algorithm takes as input the number of clusters (or classes) *K*, and outputs a partition of *Xi.e.* each state *x*∈*X* is associated with a unique class $c_{x}\in [1\ldots K]$. Next, using the known domain occurrences in the proteins of the target species and its close relatives, the match states of the original HMMs are aligned on these proteins with the Viterbi algorithm [5]. From these alignments, we count, for each state class, the number of times each amino acid is aligned on a state of this class (see Figure 1 for an example). These counts are used to compute an amino-acid distribution of the state class, which reflects how the physical and chemical constraints of this class translate into the target species. The new HMM library is built by mixing the original distribution of each match state with the estimated distribution of its class, *i.e.*:

(2) $$x^{*}=x\cdot p+a(c_{x})\cdot (1-p),$$

where *p* is the mixture proportion, and *a*(*c*_*x*_) is the amino-acid distribution associated with class *c*_*x*_. Note that while other clustering algorithms could be used, the *K*-means algorithm provides a fair tradeoff between run time (*X* involves around 470000 states to be clustered) and the quality of the results.

### Estimating the error rate of an HMM library

Evaluating and comparing HMM libraries is a difficult task. As explained above, the original Pfam library provides, with each HMM, a manually-curated score-threshold which ensures few false positives among the detected domains. One effect of the HMM-fitting approaches described above (with exception of the score-adjustment method) is to move the amino-acid composition of HMMs toward the composition of the target organism. Globally, this tends to increase the score of proteins of the organism for any HMM. Hence, the recommended thresholds cannot be safely used with the modified libraries. We propose here a simple procedure to estimate the proportion of false positives among the new domains identified with a particular library. D(ℒ,e) denotes the set of new domain occurrences identified by the library  under the E-value threshold *e*. We want to estimate the FDR associated with (ℒ,e), *i.e.* the probability *Pr*(*d*=false) for d∈D(ℒ,e).

A well known tendency of protein domains is to appear preferentially with a few other favourite domains within a protein [20]. We show here how this property can be used to estimate the FDR associated with (ℒ,e). The first step is to identify, from the whole set of annotated Uniprot proteins, domain pairs that are conditionally dependent, *i.e.* that are observed in the same proteins a significantly higher than expected number of times. This is achieved with the Fisher’s exact test, to cope with potentially small sample sizes [13]. A p-value is computed for each domain pair, and the pairs below a given threshold are stored in a set *C* of Conditionally Dependent Pairs (*CDP*). Next, from the target-species proteins that possess both known and new domains, we build a list of (known-new) domain pairs *L*, by randomly associating each new domain with one of the already known domains of the same protein. We denote as (*d*_*k*_*d*_*n*_) a pair of (known,new) domains of *L*. The list *L* is used to estimate the FDR of (ℒ,e). We assume that the proportion of false positives among the new domains *d*_*n*_ of *L* is globally the same as in all domains of D(ℒ,e). In particular, this assumes that, for a given E-value threshold, the proportion of false positives in domains of multi-domain proteins (those that are in *L*) is the same as in domains of mono-domain proteins (that are not in *L*). Although domains of mono- and multi-domain proteins are usually different, they globally share the same amino-acid composition, and there is no reason to believe that HMMs are more prone to false positives for either type.

Let |*L*| be the number of pairs in *L*. Now, we denote as  the probability that a pair in *L* belongs to the set of CDPs *C*, given that the potential domain is a true positive; similarly,  is the probability that a pair in *L* belongs to *C*, given that the potential domain is a false positive. We can express the expected number of pairs in *L* that belong to *C* as

(3) $$\begin{array}{ccc} E\left[\left|L_{C}\right|\right] & =|L|\cdot \mathrm{Pr}((d_{n},d_{k})\in C) & \\ =|L|\cdot \left(\mathrm{Pr}((d_{n},d_{k})\in C|d_{n}=\text{true})\right)\\ \times \mathrm{Pr}(d_{n}=\text{true})+\mathrm{Pr}((d_{n},d_{k})\in C|d_{n}=\text{false})\\ \left(\times \mathrm{Pr}(d_{n}=\text{false})\right)\\ =|L|\cdot \left(풯\cdot (1-\mathrm{FDR})+Ƒ\cdot \mathrm{FDR}\right). \end{array}$$

Thus, we have

(4) $$\mathrm{FDR}=1-\frac{\frac{E\left[\left|L_{C}\right|\right]}{\left|L\right|}-ℱ}{풯-ℱ}\cdot$$

|*L*| is known, and $E\left[\left|L_{C}\right|\right]$ is estimated by the observed number of pairs in *L* that belong to *C*. For , a list ^*L′*^ is created by randomly permuting new domains of the pairs in *L*. This is equivalent to randomly permuting the new domains in the proteins of the target species, and thus simulates a situation where almost all new domains are likely false positives.  is estimated by the proportion of ^*L″*^pairs that are in *C*. The procedure is repeated several times and averaged to obtain a better estimate. For , we use the known domain occurrences. A list ^*L*′^is created from all (known,known) domain pairs observed in proteins with at least two known domains. This simulates the situation where all new domains are true positives, and  is estimated by the proportion of ^*L*′^pairs that are in *C*.

One issue with expression (3) is that the estimated value may depend on the p-value threshold used to build the set of CDPs. However, in experiments on the *P. falciparum* proteome (see below), we do not observe this dependency, and standard thresholds between 1^0−2^and 1^0−4^give very similar results (see Additional file 1: Figure S1). Furthermore, it is important to note that  and  have very different estimated values. The value of  is above 99%, while that of  lies between 1% and 2%, independent of the considered library and E-value threshold (see Additional file 1: Figure S2). Hence, expression (3) could be simplified as $\mathrm{FDR}∼1-\frac{E\left[\left|L_{C}\right|\right]}{\left|L\right|}$. Based on this equation, the FDR is simply equal to the proportion of domain pairs in *L* which do not belong to the set *C*. In other words, almost every correct new-domain in *L* is in a pair that is in the set *C*, and a new domain from a pair that is not in *C* is most likely incorrect. This astonishing fact clearly illustrates the strong correlations that exist between domains in multi-domain proteins. It also reveals that almost all the correlations between domains can be deduced from the set of Uniprot proteins, *i.e.* most domain associations have already been observed a significantly high number of times.

Another issue is the statistical error induced by the sample *L* in our FDR estimate. We address this issue with a classical bootstrap procedure [21], in which a bootstrapped list *L*_*b*_ is build by randomly sampling with replacement |*L*| pairs of *L*. From this list, we compute a new FDR estimate *FDR*_*b*_using the procedure described above—*i.e.* we compute a new estimate of  and $E\left[\left|L_{C}\right|\right]$—, and the entire procedure is repeated a large number of times *B* (for example, *B*=500). We then have a sample of *B* independent bootstrap replications of the FDR estimate, and we use the standard deviation of this sample as an estimate of the standard error.

## Results

We applied the four approaches described above—*i.e.* Score adjustment, Enriched alignment, Substitution matrix and Match-state clustering—to the proteome of *P. falciparum* (PlasmoDB release 5.5), using the Pfam HMM library (version 23.0). There are a total of 5460 proteins in *P. falciparum*, 2900 of which have a known domain occurrence. In our substitution matrix approach, we must choose a stationary distribution. We tried two solutions for this. The first one corresponds to the average amino-acid distribution of *P. falciparum* proteins. However, *P. falciparum* proteins are known to exhibit low-complexity inserts with very biased amino-acid distribution which are responsible for an average increase of 20% in the total length of the proteins [9]. These low-complexity segments are usually found between domains, but may also reside within a domain. Following Pizzi and Frontali [9], we used the SEG algorithm [22] to remove low-complexity segments and to compute an amino-acid distribution of high-complexity regions which is utilized as an alternative stationary distribution. For the time parameter *t*, which represents the mutation probability at each position, we tried seven values: 0.01, 0.05, 0.1, 0.2, 0.3, 0.4, and 0.5. Finally, for the match-state clustering approach, different numbers of classes ranging from *K*=50 to *K*=500, as well as three mixture proportions (25%, 50%, 75%) were tested.

For each correction method and parameter value, a new HMM library is built. Each library is then calibrated with exception of the score-adjustment method derived library. Calibration is achieved by fitting the Gumbel distributions used for E-value computations to the new models (see HMMER 2.3.2 User’s Guide [14]). After this step, each library is used to search for new domain occurrences below a given E-value threshold in *P. falciparum* proteins. In the following, we use the modified libraries for two problems. First, the aim is to identify new domains with low E-values; Second, the aim is to identify more divergent domain occurrences (with higher E-values) with the help of the CODD procedure we have recently proposed [13].

### Results for low E-value occurrences

The new libraries were first used to find new domains at low E-value (from 1^0−3^ to 1^0−1^). For each library, the FDR associated with the newly identified domains was estimated with the procedure described above.

In a first experiment, we used all known domains of available *Alveolata* proteins to fit the HMMs. This includes domains from 13 completly sequenced *Apicomplexa* species—six *Plasmodium*, three *Cryptosporidium*, two *Theileria*, *Babesia bovis* and *T. gondii*—, the ciliate *Paramecium tetraurelia*, and numerous translated ORFs of isolated *Alveolata* sequences. For the score-adjustment approach, the six *Plasmodium* species are in the genus (denoted *S*_1_in formula (1)), the two *Theileria* species and *Babesia bovis* are in the *Aconoidasida* class (*S*_2_), the three *Cryptosporidium* species and *T. gondii* are in the *Apicomplexa* phylum (*S*_3_), and the kindom (*S*_*m*_) incorporates all eukaryotes.

**Figure 2:**
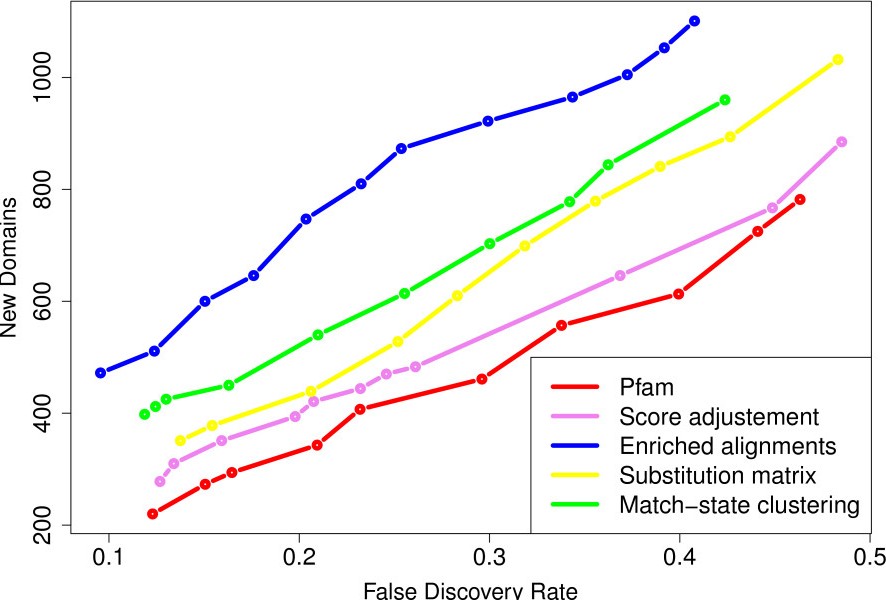
**Sensitivity and accuracy of the four correction approaches, using the known domains of all available *Alveolata*.** Number of new domains (y-axis) identified for a given FDR (x-axis).

The Additional file 1: Figure S3 shows the results achieved by the substitution matrices and match-state clustering approaches for different parameter values. For the substitution-matrices approach, removing low-complexity regions before computing the stationary distribution increases the number of new domains, at equivalent error rate. As for the time parameter, the best results are achieved with *t*=0.1. For match-state clustering, the curves suggest that 75% is the best mixture proportion. At low FDRs, the differences between the performance achieved by the different values of *K* are small, but *K*=100 seems to be the best. The investigated parameter values were chosen to span relatively large and *a priori* sound intervals. However, a more systematic exploration of the parameter space—especially for the time parameters *t* and the mixture proportion *p*—may slightly improve the results. However, we have chosen not to embark further on the parameter space exploration, as expected improvements are likely moderate, and as this allows for a fair comparison with the score-adjustment and enriched-alignment methods which both lack parameters. Figure 2 compares the best results achieved by the four approaches when using the known domains of *Alveolata* species. For comparison, we also show the results achieved with the original Pfam library at the same E-value thresholds (*i.e.* from 1^0−3^ to 1^0−1^). All methods discover several additional domains not identified with the standard library. The enriched alignment approach (blue curve) achieves the best results. Even if this approach cannot reconstruct all HMMs, the reconstructed models correspond to the most frequent families in the taxon. The match-state clustering approach (green curve) and substitution matrices (yellow curve) also identify additional domains. The weaker results obtained by the latter method may be due to the standard substitution schema that was employed. Indeed, exchangeability matrices model “universal” evolutionary mechanics, while *P. falciparum* is constrained by more extreme evolutionary circumstances. Moreover, this is the only approach that does not use information from close species. Finally, the score correction approach also discovers some domains not identified with the original Pfam library with the standard scoring function.

**Figure 3:**
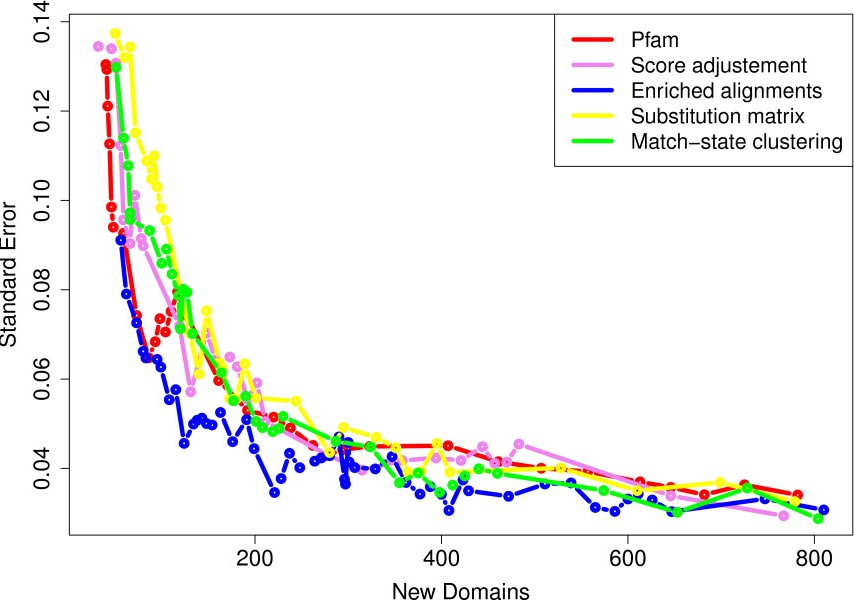
**FDR standard error of the four correction approaches.** FDR standard error (y-axis) estimated for a given number of new domains (x-axis).

**Figure 4:**
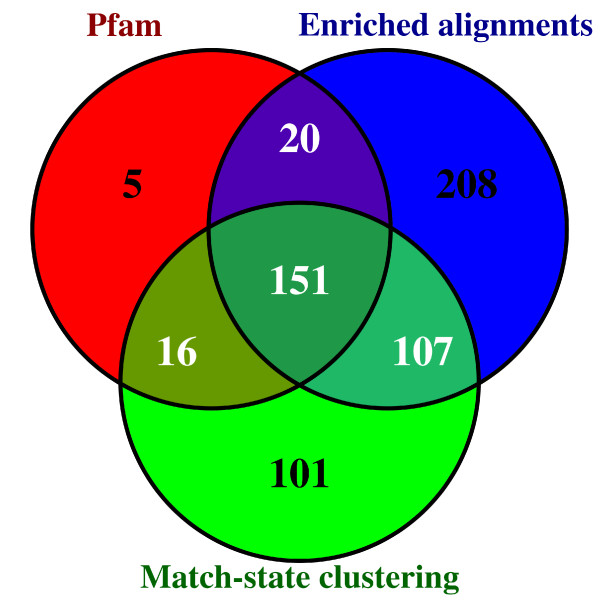
**Number of new domains identified at 10% FDR.** Number of new domains identified by the Pfam standard library when relaxing the E-value threshold (red), the match-state clustering approach (green), and the enriched alignment approach (blue).

We also estimated the standard error of the FDR estimate using the bootstrap procedure previously described. Figure 3 illustrates the FDR standard-error with respect to the number of newly identified domains for the different libraries. It shows that when the number of new domains is above 200 (*i.e.* for all points in Figure 2), the standard error ranges from 3% (for the largest sets) to 5% (for the smallest ones). However, when the number of new domains is smaller, the FDR standard-error increases, and the FDR estimate become unreliable. To understand this sudden increase in standard error, one must recall that the FDR is estimated on the basis of a subset of the new domains—those that are in a protein where a domain is already known. Hence, when the number of new domains is around 200, the number of domains actually used in the FDR estimate is between 55 and 60 depending on the library. Figure 4 summarizes the predictions achieved with the enriched alignment, match-state clustering and Pfam standard library approaches at 10% FDR. We observe that the corrected libraries include most of the domains also identified with the standard Pfam library when relaxing the E-value thresholds. On the contrary, the domains solely identified by the corrected libraries are more diverse, and many new predicted occurrences are specific to one of the approaches. All results obtained with the different libraries at 10% FDR are summarized in Table 1 (see Additional file 1: Table S1 in for results at 20% FDR). The results suggest that although the enriched-alignment approach identifies the largest number of new domain occurrences, the match-state clustering approach provides close results in terms of domain diversities (243 *vs.* 283 at 10% FDR), and identifies slightly more domain families that were previously unknown in the *Apicomplexa* phylum (40 *vs.* 15).

**Table 1:** New domains in *P. falciparum* at 10% FDR

	Dom.	Fam.	Abs. P.f.	Abs. Alv.
Pfam	192	132	60	17
Score adjustment	239	159	67	20
Enriched alignment	486	283	101	15
Substitution matrix	228	147	54	18
Match-state clustering	375	243	103	40

Number of new domains (Dom.), domain families of the new domains (Fam.), domain families previously thought to be absent in *P. falciparum* (Abs. P.f.), and domain families previously thought to be absent in all Alveolata (Abs. Alv.), identified by the correction approaches and the standard Pfam library at 10% FDR.

To further assess the newly discovered domains, we next looked at the other InterPro domains that do not belong to the Pfam database. The InterPro database incorporates several domain databases (PROSITE, PRINTS, ProDom, SMART, TIGRFAMs, PIRSF, SUPERFAMILY, Gene3D, PANTHER and Pfam) into a single resource. This meta-database organizes entries in *InterPro families* that pool all representations of the same domain [3]. The different databases behind InterPro use their own domain definition and representation, and thus present a heterogeneous view of protein domains. Taken altogether, predictions of these different databases are more sensitive than that of the Pfam database only, but they may also be less accurate. Table 2 reports the number and proportion of newly identified Pfam domains (at 10% FDR) that belong to an already known InterPro family in the same protein. For comparison purpose, we also computed the proportion of matches achieved on random domains, by randomly permuting the new domains across proteins (and repeating the procedure 1000 times to obtain accurate estimates). For every HMM-fitting procedure, around 50% of new domains match with a known InterPro entry of the protein. This is a high proportion compared with what is expected on random domains (around 2%), which indicates that this 50% domains are likely true positives. Moreover, although the entire InterPro database is more complete than the Pfam database only, it is far from being exhaustive. Hence, this result does not imply that the remaining ∼50% new domains that do not belong to a known InterPro family in the same protein are false positives.

**Table 2:** InterPro coverage of new domains at 10% FDR

	InterPro Cov.	InterPro Cov. H0
Pfam	103 (53.9%)	3.34%
Score Adjustment	129 (54.2%)	2.85%
Enriched Alignment	230 (47.9%)	1.70%
Substitution Matrix	121 (53.5%)	3.06%
Match-state Clustering	191 (52.2%)	1.84%

InterPro Cov.: Number and proportion (in parenthesis) of new domains belonging to an InterPro family previously known for the protein, for the different correction approaches and the standard Pfam library, at 10% FDR; InterPro Cov. H0: proportion of matches on random domains.

**Figure 5:**
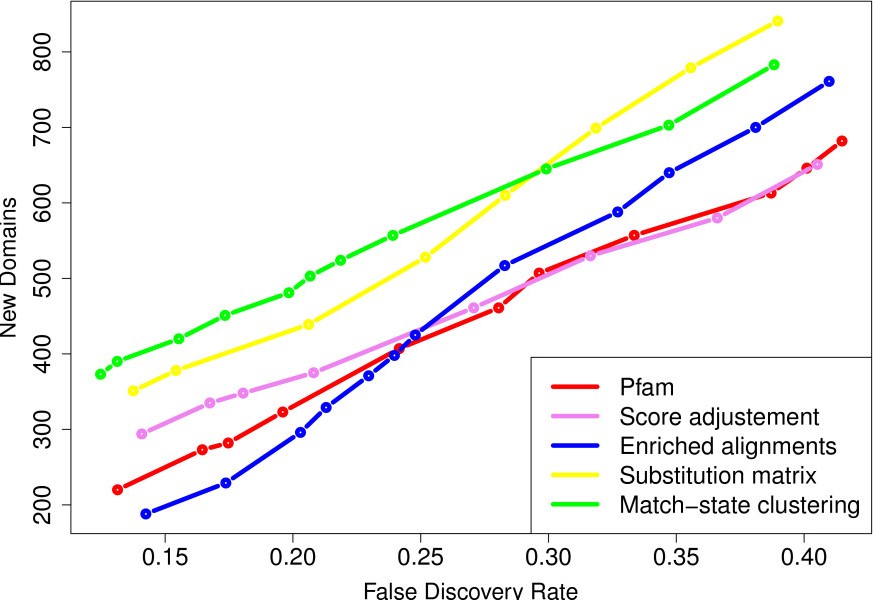
**Sensitivity and accuracy of the four correction using only known domains of *P. falciparum*.** Number of new domains (y-axis) identified for a given FDR (x-axis).

Apart from the substitution-matrix method, all approaches make use of the already known domains of *P. falciparum* and of the other available *Alveolata* species. To compare the performance of the methods depending on whether species close to the target were available or not, we conducted a second series of experiments using only known Pfam domains of the *P. falciparum* genome to fit the libraries (see Figure 5). For the score-adjustment approach, this involves using only two levels: *S*_0_ for *P. falciparum*, and *S*_1_for the kingdom (all eukaryotes). In these conditions, the results strongly differ from the previous ones. The number of new domains identified by the enriched alignment approach drops off, and even passes below the number of domains identified by the original Pfam library at low FDR. On the contrary, the match-state clustering approach still provides numerous additional domains. With the substitution-matrix approach, it appears to be the best approach when no close species are available. While for *P. falciparum* the practical interest is limited, this illustrates the potential of such approaches for all genome sequencing projects of organisms from poorly known phyla, with no already sequenced close-species.

### Results for more divergent occurrences

We next investigated the performance of the modified libraries for identifying divergent domain occurrences using the CODD procedure we have recently proposed [13]. CODD improves the sensitivity of HMM domain detection by directly exploiting the co-occurrence domain tendency used in the FDR estimation method described above. Given a set of new domain occurrences below a permissive E-value threshold (and thus with a high number of false positives), CODD selects those that form, together with another domain of the same protein, a pair previously identified as being conditionally dependent (*i.e.* a pair of the CDP set). The domains selected this way are said to be *certified*. The certification can be done on the basis of the already known Pfam domains of the protein, but also on the basis of the other known InterPro (non Pfam) domains, or even on the basis of the other new Pfam domains that are below the E-value threshold. Moreover, CODD uses a shuffling procedure to provide its own estimate of the FDR associated with the certified domains [13]. Because domain co-occurrence is a strong indicator of real occurrence, CODD can certify the presence of a domain with low FDR even for very high E-values. In the following, it is used to certify the presence of domains with E-values up to ten, far higher than the highest E-values considered in the previous section (1^0−1^). As CODD also certifies the presence of domains with low E-values, part of the certified domains are already considered in the previous section. However, note that CODD is obviously limited to the certification of domains from multi-domain proteins, and thus that a large part of the previously considered domains (around 75%) cannot be certified. Moreover, it is worth noting that, contrary to the previous FDR, which involves all domains below a given E-value threshold, the FDR estimated by CODD only concerns the certified domains.

**Figure 6:**
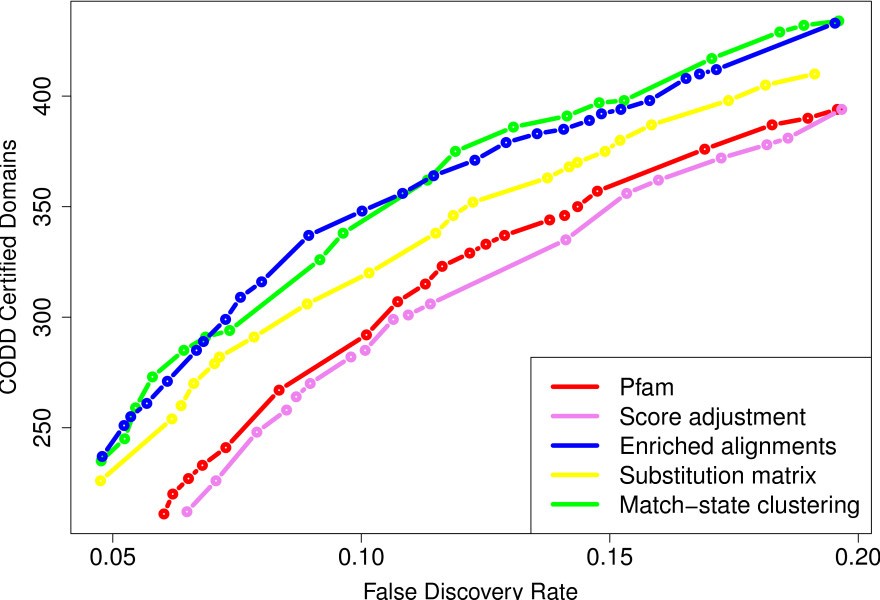
**Sensitivity and accuracy of CODD on the four corrected libraries.** Number of new domains (y-axis) identified by CODD for a given FDR (x-axis), using the already known Pfam domains for the certification.

Figure 6 summarizes the results achieved by CODD with the original and modified libraries. E-value threshold were varied from 1^0−1^ to 10, and the already known Pfam domains were used for the certification. In this experiment, the score adjustment, enriched alignment and match-state clustering approaches make use of the known domain occurrences of all *Alveolata*. The results differ somewhat from those achieved at low E-value in the same conditions. The enriched alignment and match-state clustering approaches now achieve similar sensitivity, while the score-adjustment approach does not detect more new domains than the standard Pfam library. This can be explained by the fact that the high E-value domains identified by co-occurrence are often uncommon among the already known domains of the species [13,23]. This likely affects the performance of methods that strongly rely on known domain occurrences.

As with low e-value thresholds, the substitution-matrix approach outperforms the original Pfam library, but does not achieve as good results as the match-state clustering approach. It is worth noting that the stationary distribution used in these experiments—*i.e.* the one obtained when removing low-complexity regions from *P. falciparum* proteins—is closer to the classical equilibrium amino-acid frequencies (used in WAG for example [16]) than the global *P. falciparum* distribution is. Thus, for comparison, we also built different HMM libraries using the WAG equilibrium frequencies as stationary distribution. The results achieved with different time parameter values are in Additional file 1: Figure S4. Although the WAG equilibrium frequencies never achieved results as good as the *P. falciparum* distribution without low-complexity regions, it also outperforms the standard Pfam library. This seems to indicate that simply smoothing the match-state distribution of the HMMs is an adequate strategy for increasing the number of discovered domains in divergent organisms like *P. falciparum*.

We also applied a bootstrap procedure similar to that described above to estimate the standard error associated with the CODD FDR. Irrespective of the library, the standard error remains quite low, ranging from 0.5% for the highest FDRs to 1.5% for the smallest ones (data not shown). Table 3 reports the results achieved by CODD on the different libraries at 10% (see Additional file 1: Table S2 for results at 20% FDR). Note that contrary to Figure 6 which only reports the number of domains certified with the known Pfam domain occurrences, this table combines the certifications achieved with all known domain occurrences (*i.e.* Pfam and non-Pfam) as well as with the other new domain occurrences below the considered threshold.

**Table 3:** New CODD domains in *P. falciparum* at 10% FDR

	Dom.	Fam.	Abs. P.f.	Abs. Alv.
Pfam	404 (330)	228 (169)	85 (66)	26 (24)
Score Adjustment	427 (332)	226 (151)	82 (63)	24 (22)
Enriched Alignment	529 (312)	274 (131)	93 (55)	21 (18)
Substitution Matrix	474 (379)	266 (197)	99 (85)	32 (31)
Match-state Clustering	516 (358)	288 (180)	111 (81)	39 (34)

Number of new domains (Dom.), domain families of the new domains (Fam.), domain families previously thought to be absent in *P. falciparum* (Abs. P.f.), and domain families previously thought to be absent in all *Alveolata* (Abs. Alv.), identified by CODD with the different libraries at 10% FDR. Numbers in parenthesis refer to domains/families that are new compared to Table 1 for the same library.

### Annotation of *P. falciparum* proteins

Any item or feature that would help assigning a function to a gene is precious for biologists. We thus investigated GO annotations that could be deduced from all newly identified domains. As described in the introduction, some domains have been associated with specific GO terms by the InterPro consortium. The policy is to associate, with a given domain, annotations shared by all annotated proteins possessing this domain. Moreover, by extending this policy to domain combinations (as described in [24]), several additional GO terms can be deduced from the combination of two or more domains. To this end, we enumerated all Pfam domain combinations in the proteins of Swiss-Prot, and identified, for each combination, the GO terms shared by all annotated proteins where the combination is present (only combinations observed in at least ten annotated proteins were considered). We found 2235 Pfam domain combinations associated with at least one specific GO annotation: 2115 domain pairs, 119 domain triplets and 1 quartet. All associations between domain combinations and GO terms are available at http://www.lirmm.fr/∼terrapon/HMMﬁt/. Altogether, single domains and domain combinations improve the annotations of several *P. falciparum* proteins. Table 4 gives the number of new annotations brought by the new domains identified at low E-values or with the help of the CODD procedure at 10% FDR (see Additional file 1: Table S3 for results at 20% FDR). For example, the new domains identified with the match-state clustering library leads to the discovery of 355 new GO annotations, *i.e.*∼6*%* of the 5791 already known GO annotations of this organism. All predictions on *P. falciparum* achieved using the *Alveolata* species have been integrated into a dedicated website which gathers all known and newly discovered domains and GO annotations for the proteins of this organism. The site provides details on E-values, FDRs and alignments, and includes useful links to Pfam, InterPro and PlasmoDB websites. Moreover, when a new domain has been certified with CODD, this information, along with details of the certification process, are included on the website.

**Table 4:** New GO annotations of *P. falciparum* proteins at 10% FDR

	Pfam	Score	Enriched	Substitution	Match-state
		correction	Alignments	matrices	clustering
New GO	268	268	348	307	355
Unan. prot.	32	36	48	35	51

Number of new GO annotations brought by the different correction methods and by the original Pfam library at 10% FDR. “New GO” is the total number of GO annotations, and “Unan. prot.” is the number of proteins without known annotation for which an annotation has been proposed.

Although the expected number of false positives in predictions with 10% FDR is low, it is not equal to zero. Hence, an expert examination of the predictions is recommended to identify the 10% false positives. This can be done in several ways: by looking at the predictions shared with the other corrected libraries, by checking if a prediction agrees with known protein annotations (*i.e.* known domains, known functional annotations, protein attributes, etc.), and by carefully looking at the protein alignments.

Among the predictions, several putative domains have attracted our attention as they might provide insights into important biological functions essential to understand the biology of the parasite, including the control of gene expression, the mechanisms of cell proliferation, the biogenesis of intracellular organelles and the parasitic metabolism. Hereafter, we give a few examples that have been identified using the CODD procedure on the modified libraries.

Regarding the control of gene expression, it is now established that an important general process in this parasite involves the control of chromatin condensation via chemical modifications of histones and/or of DNA bases [25]. PFD0840w encodes a conserved hypothetical protein in which we have detected, with the match-state clustering library, a DNA binding and a DNA methylation domain that would make this protein an important actor in the control of the genetic expression in *Plasmodium*. In the same context, the putative role of PFB0290c in the control of gene transcription is further consolidated by the detection of domains functionally associated with DNA binding and transcription from RNA polymerase III promoters (identified by match-state clustering and substitution-matrix libraries).

The paucity of genes involved in important metabolic pathways has also been highlighted in a series of *in silico* analyses of the malaria genome (see for example [26]) and expectations in the reannotation with respect to the discovery of missing or novel metabolic enzymes are high. A striking feature is the apparent lack of glycosyltransferases (only eight recorded in the CAZy database [27]), suggesting that this eukaryote would only catalyze eight hexosyl-transfer reactions and only one glycosyl-hydrolase. This lends support to the notion that a parasitic lifestyle does not require the utilization of exogenous linked sugar sources. Here, we identified domains in the MAL13P1.66 protein sequence that suggest a possible interaction with carbohydrates, including the transport of sugars and/or the catalysis of a glycosyl-transfer similar to that catalyzed by enzymes of the glycosyltransferase family 1 of the CAZy classification (identified by match-state clustering and substitution-matrix libraries). Future experimental studies of the corresponding gene and proteins should be conducted as MAL13P1.66 may prove to be an important protein in the carbohydrate metabolism of *P. falciparum*.

Regarding the biogenesis of intracellular organelles, the vital importance of the apicoplast has stimulated numerous studies geared at functionally dissecting the import and maturation of nuclear encoded proteins (for review see [28]). PF14_0249, which encodes a hypothetical protein of the apicoplast previously reported as possibly associated with the organelle membrane, is suspected to harbour a peptidyl-prolyl cis-trans isomerase activity by the occurrence of domains Trigger_C (PF05698) and FKBP_C (PF00254) (match-state clustering and substitution-matrix libraries). It could therefore play an important role in the accurate folding of imported proteins when they reach stroma of the apicoplast. These examples illustrate how the detection of novel domains might be helpful for further studies of this essential, yet poorly understood, parasite proteome.

## Discussion and Conclusions

We have proposed two new methods to fit an HMM library to a target organism. These methods have been implemented in a software freely available at http://www.lirmm.fr/∼terrapon/HMMﬁt/. Our methods learn global correction rules that are applied to match states of the entire library, thus enabling the discovery of domains that were previously unknown in a given organism. The two methods concentrate on modification of the amino-acid distribution of match states. This is because match-states model conserved positions of the proteins, and thus likely contain most of the information of the sequence alignments. However, several other solutions can be explored, such as modifying the number of states, the probabilities of between-state transitions, the amino-acid distributions associated with insertion states, or the null model used for computing scores and E-values.

Additionally, we have presented a simple procedure to estimate the proportion of false positives in a set of newly identified domains. Using *P. falciparum* as a case study, we assessed the performances of our methods and of two previous approaches that rely on a specific adjustment of the score function, or on the enrichment of sequence alignments. Note that several other approaches address related issues. For example, Kumar and Cowen [29] propose to augment the sequence alignments with artificial sequences generated by simulated evolution. Similarly, several studies propose to improve the training of profile HMMs and the scoring of sequences using negative examples (e.g. see [30-32]). However, the aim of these methods differs from ours. Instead of fitting HMMs to a particular species, their goal is to distinguish the different sub-families within a large protein family. Consequently, we have not considered these studies further in the presented work.

In *P. falciparum*, our experiments show that when several close genomes are available, the approach which integrates known domain occurrences into the sequence alignments provides the best results in terms of number of new occurrences. Although our approach based on match-state clustering identifies fewer new domains, it provides similar results in terms of domain diversity, and discovers slightly more new domain families. Moreover, when there is no closely related species available, our two approaches outperform the other methods in terms of occurrence number, domain diversity and number of previously unknown families. This is also the case when the modified libraries are used to detect divergent domain occurrences with the help of the CODD procedure.

In summary, the fitted libraries identify in *P. falciparum* several hundred domains that are not identified with the original Pfam library. At low E-value thresholds, the Pfam standard library identifies 192 new domains at 10% FDR, while the enriched alignment and match-state clustering libraries identify 486 and 375 new domains, respectively, at the same FDR. With the help of the CODD procedure, the original library identifies 404 new domains at 10% FDR, while the enriched alignment and match-state clustering libraries identify 529 and 516 new domains, respectively. Additionally, the newly identified domains often provide new GO annotations for *P. falciparum* proteins. For example, the enriched alignment and match-state clustering libraries lead to an additional 348 and 355 new GO annotations, respectively. All new domains discovered on *P. falciparum* have been integrated into a dedicated website which pools all known/new annotations of protein domains and functions for this organism.

## Competing interests

The authors declare that they have no competing interests.

## Authors’ contributions

NT, OG and LB conceived and designed the method and experiments. NT implemented the approach. EM analysed the biological results. NT and LB drafted the manuscript. OG initiated the project. All authors have read and approved the final manuscript.

## Supplementary Material

Addtional file 1 **Supplementary figures and tables.** This PDF file contains four supplementary figures, and three supplementary tables. Click here for file
