# Supplementary material for: Fitting hidden Markov models of protein domains to a target species: application to Plasmodium falciparum
Source: BMC Bioinformatics. 2012 May 1;13:67. doi: 10.1186/1471-2105-13-67 (PMC3434054; doi:10.1186/1471-2105-13-67)
Supplement: Addtional file 1 — Supplementary figures and tables. This PDF file contains four supplementary figures, and three supplementary tables. [file 1471-2105-13-67-S1.pdf]

Fitting hidden Markov models of protein domains to a target species: application to *Plasmodium falciparum* Supplementary Figures and Tables

Nicolas Terrapon[1,2], Olivier Gascuel[1], Éric Maréchal[3] and Laurent Bréhélin[1]

[1]Méthodes et algorithmes pour la Bioinformatique, LIRMM, Univ. Montpellier 2, CNRS, 161 rue Ada 34392 Montpellier Cedex 5 France
[2]Institute für Evolution and Biodiverstät – Westfälische Wilhelms-Universität, Hüfferstrasse 1, D48149 Münster, Germany
[3]CEA Grenoble iRTSV/LPCV, 17 rue des Martyrs, 38054 Grenoble cedex 9 France

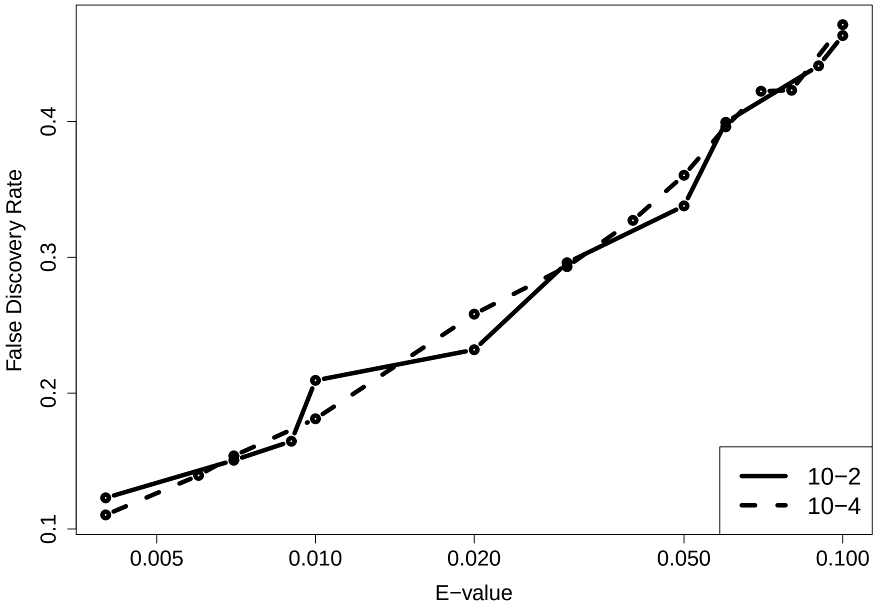

Supplementary Figure 1 — Effect of the p-value threshold used for CDP selection on the FDR estimate. This figure reports the estimated FDR (y-axis) associated with new domains identified below a given E-value threshold (x-axis) for the original Pfam library. Plain and dashed lines report the FDRs estimated with a CDP list built with a p-value threshold of 10^{-2} and 10^{-4}, respectively.

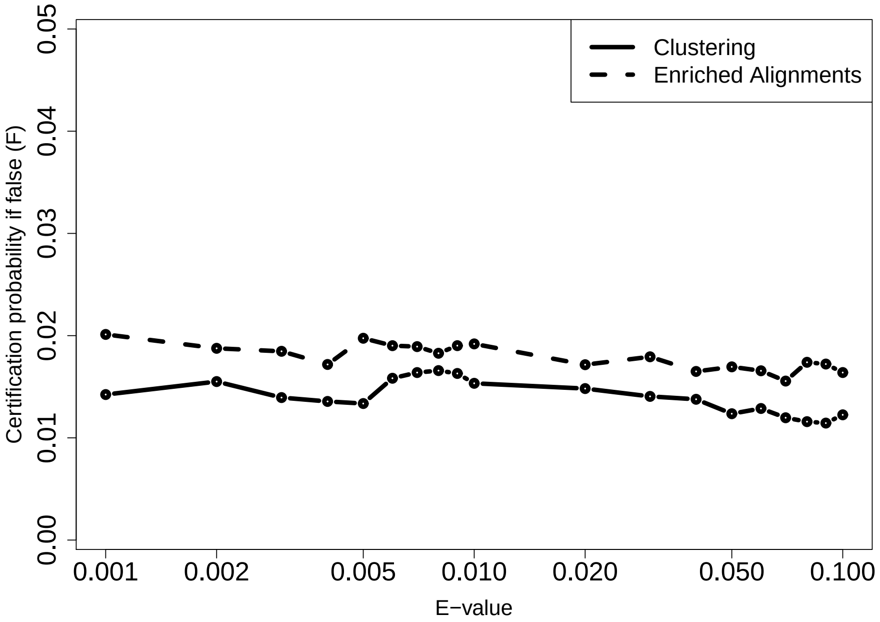

Supplementary Figure 2 — $\mathcal{F}$ estimates. $\mathcal{F}$ values estimated for enriched alignment and match-state clustering approaches, for different E-value thresholds.

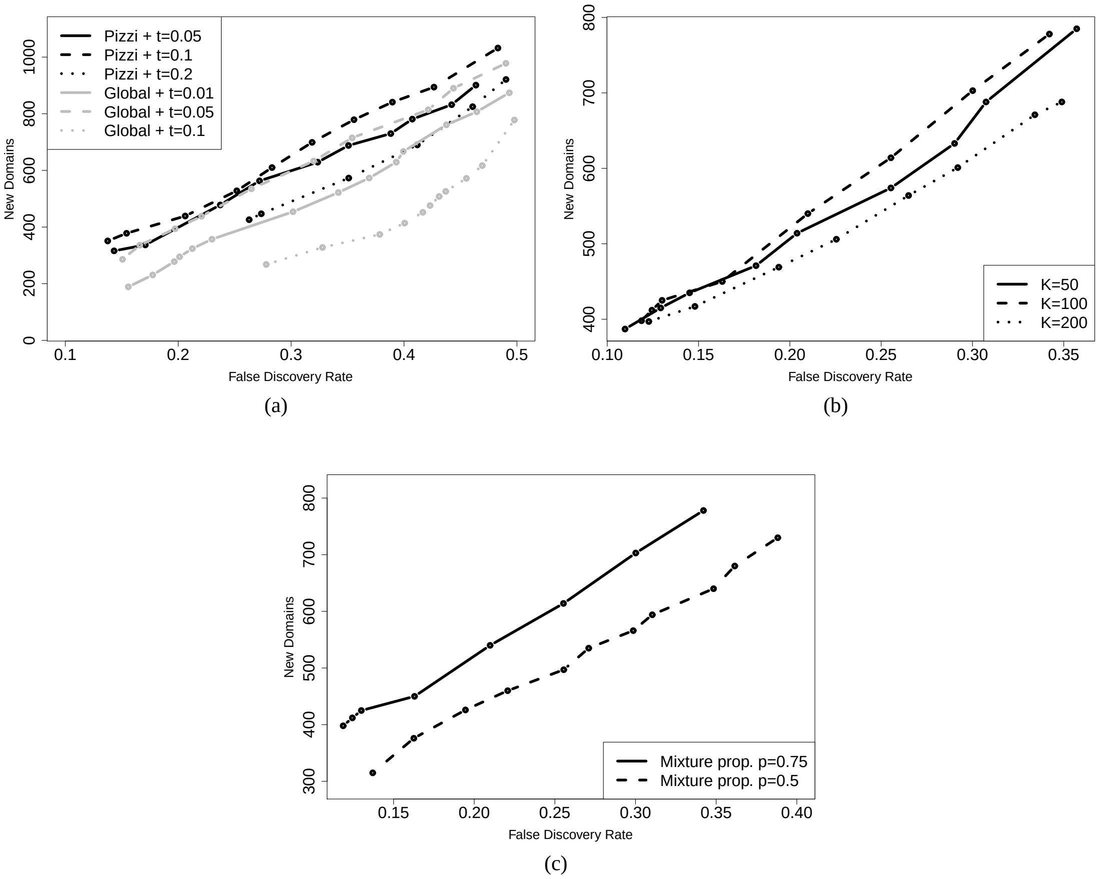

Supplementary Figure 3 — Effect of parameter values on the sensitivity and accuracy of the different correction methods. Number of new domains (y-axis) identified for a given FDR (x-axis). For the substitution matrix approach (figure 3(a)), we report the results achieved when considering the global amino-acid distribution of *P. falciparum* (grey curves) or the distribution without low-complexity regions (black curves) as stationary distribution. For the match-state clustering approach, figure 3(b) shows the effect of the number of clusters: $K = 50$ (continuous curve), $K = 100$ (dashed curve) and $K = 200$ (dotted curve), while Figure 3(c) shows the effect of the mixture proportion—p=50% (dashed line), p=75% (continuous line).

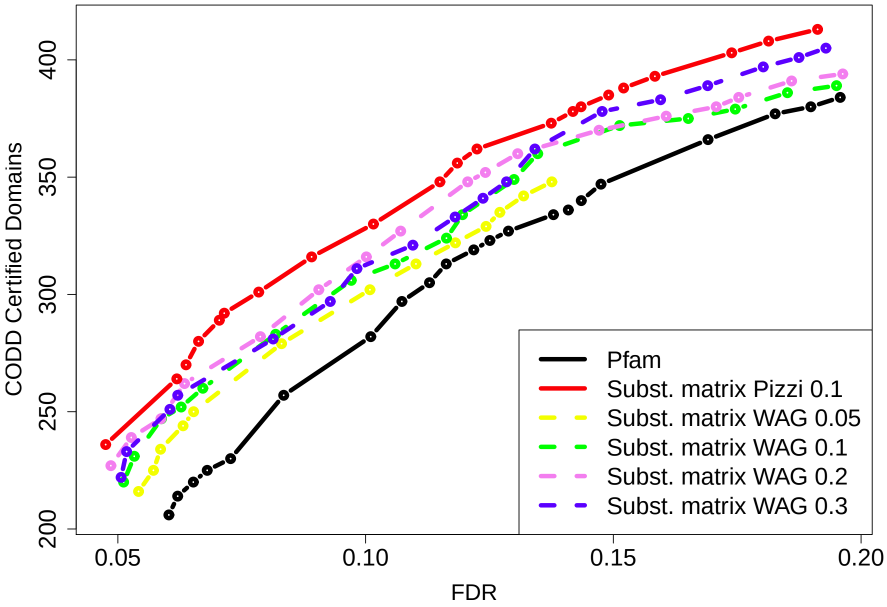

Supplementary Figure 4 — *P. falciparum* vs WAG stationnary distribution. Number of domains (y-axis) certified with CODD for a given FDR (x-axis), when using the WAG equilibrium frequencies as stationary distribution in the substitution matrix approach, for different time parameter values. For comparison, the figure also reports the results achieved with the original Pfam library, and the library obtained using the *P. falciparum* distribution without low complexity regions (Pizzi) and time parameter 0.1.

	Dom.	Fam.	Abs. P.f.	Abs. Alv.
Pfam	311	203	92	35
Score adjustment	397	248	110	44
Enriched alignment	707	402	166	46
Substitution matrix	431	267	118	51
Match-state clustering	548	338	153	68

Supplementary Table 1 — New domains on *P. falciparum* at 20% FDR. Number of new domains (Dom.), domain families of the new domains (Fam.), domain families previously thought to be absent in *P. falciparum* (Abs. P.f.), and domain families previously thought to be absent in all Alveolata (Abs. Alv.), identified by the correction approaches and the standard Pfam library, at 20% FDR.

	Dom.	Fam.	Abs. P.f.	Abs. Alv.
Pfam	701 (569)	346 (253)	155 (126)	68 (64)
Score Adjustment	700 (532)	331 (217)	149 (114)	63 (56)
Enriched Alignment	787 (499)	368 (178)	137 (80)	45 (39)
Substitution Matrix	758 (588)	387 (270)	168 (135)	79 (72)
Match-state Clustering	758 (537)	389 (238)	177 (130)	86 (77)

Supplementary Table 2 — New CODD domains on *P. falciparum* at 20% FDR. Number of new domains (Dom.), domain families of the new domains (Fam.), domain families previously thought to be absent in *P. falciparum* (Abs. P.f.), and domain families previously thought to be absent in all *Alveolata* (Abs. Alv.), identified by CODD with the different libraries, at 20% FDR. Numbers in parenthesis refer to the domains/families that are new compared to Supp. Table 1 for the same library.

	Pfam	Score correction	Enriched Alignments	Substitution matrices	Match-state clustering
New GO	438	444	587	501	535
Unan. prot.	51	57	87	58	81

Supplementary Table 3 — New GO annotations of *P. falciparum* proteins at 20% FDR. Number of new GO annotations brought by the different correction methods and by the original Pfam library at 20% FDR. "New GO" is the total number of GO annotations, and "Unan. prot." is the number of proteins without known annotation for which an annotation has been proposed.
